# The Connexin46 Mutant, Cx46T19M, Causes Loss of Gap Junction Function and Alters Hemi-channel Gating

**DOI:** 10.1007/s00232-014-9752-y

**Published:** 2014-11-18

**Authors:** Jun-Jie Tong, Peter J. Minogue, Matthew Kobeszko, Eric C. Beyer, Viviana M. Berthoud, Lisa Ebihara

**Affiliations:** 1 Department of Physiology and Biophysics, Chicago Medical School, Rosalind Franklin University of Medicine and Science, 3333 Green Bay Road, North Chicago, IL 60064 USA; 2 Department of Pediatrics, University of Chicago, 900 E. 57th Street, Chicago, IL 60637 USA

**Keywords:** Cataract, Lens, Gap junction, Hemi-channel, Cx46

## Abstract

An N-terminal mutant of connexin46 (T19M) alters a highly conserved threonine and has been linked to autosomal dominant cataracts. To study the cellular and functional consequences of substitution of this amino acid, T19M was expressed in *Xenopus* oocytes and in HeLa cells. Unlike wild-type Cx46, T19M did not induce intercellular conductances in *Xenopus* oocytes. In transfected HeLa cells, T19M was largely localized within the cytoplasm, with drastically reduced formation of gap junction plaques. Expression of rat T19M was cytotoxic, as evidenced by an almost complete loss of viable cells expressing the mutant protein by 48–72 h following transfection. When incubated in medium containing physiological concentrations of divalent cations, T19M-expressing cells showed increased uptake of DAPI as compared with cells expressing wild-type Cx46, suggesting aberrant connexin hemi-channel activity. Time-lapse and dye uptake studies suggested that T19M hemi-channels had reduced sensitivity to Ca^2+^. Whole cell patch clamp studies of single transfected HeLa cells demonstrated that rat T19M formed functional hemi-channels with altered voltage-dependent gating. These data suggest that T19M causes cataracts by loss of gap junctional channel function and abnormally increased hemi-channel activity. Furthermore, they implicate this conserved threonine in both gap junction plaque formation and channel/hemi-channel gating in Cx46.

**Electronic supplementary material:**

The online version of this article (doi:10.1007/s00232-014-9752-y) contains supplementary material, which is available to authorized users.

## Introduction

Studies of mutants identified by linkage to diseases or by systematic site-directed mutagenesis are helping to clarify the function and regulation of channels and hemi-channels formed by connexins (Cx). The connexins are a family of proteins that form gap junction plaques which contain intercellular channels that directly connect the cytoplasm of one cell with that of a neighbor. Gap junctional channels are formed by the docking of two hexameric connexin assemblies (connexons), contributed by the two communicating cells. Undocked connexons are also present in the non-junctional plasma membrane where they can act as transmembrane “hemi-channels” that are gated by extracellular divalent cations, intracellular concentrations of Ca^2+^ and H^+^, and voltage (Ebihara 2003; Sáez et al. 2010).

The connexin family contains 21 human members that share extensive sequence identity and similarity (Beyer and Berthoud 2009). All of the connexins have a similar transmembrane topology, containing four transmembrane domains with both the N- and C-termini residing on the cytoplasmic side of the membrane. The N-terminus makes a substantial contribution to the channel properties. Structural studies of another family member, Cx26, have shown that the N-terminus inserts into the channel pore and forms a funnel (Maeda et al. 2009). Functional studies of several connexins indicate that alterations of charged residues in this domain affect channel gating and permeability (Dong et al. 2006; Oh et al. 2000, 2008; Purnick et al. 2000; Tong et al. 2004, 2013; Tong and Ebihara 2006; Verselis et al. 1994).

Connexin mutations have been linked to a variety of different pathologies including deafness, X-linked Charcot-Marie-tooth disease, skin diseases, oculodentodigital dysplasia, and cataracts. Our laboratories have particularly focused on examining the cellular and physiological abnormalities caused by mutations of Cx46 and Cx50 that are associated with inherited congenital cataracts. These studies have identified various mechanisms by which the mutations lead to disease, including loss-of-function, gain-of-(hemi-channel)-function, and dominant-negative inhibition of wild-type connexin function (Beyer et al. 2013).

The present study was designed to examine the cellular and physiological behavior of a recently identified Cx46 mutant that causes the replacement of the threonine at position 19 with methionine (T19M). This mutation was identified in members of a family of people who developed posterior cortical cataracts that were inherited in an autosomal dominant manner (Santhiya et al. 2010). Threonine-19 is a residue that is nearly perfectly conserved in all members of the connexin family, implying the importance of this amino acid for connexin function. However, there have not been previous systematic studies of disease-linked substitutions at this position. Therefore, elucidation of the abnormalities conferred by this mutation may yield insights into the pathogenesis of inherited cataracts and diseases linked to other connexins mutated at this position.

The data presented below show that the T19M substitution results in alterations of gap junction channel and hemi-channel behaviors.

## Materials and Methods

### Generation of Cx46 Constructs

DNA segments encoding rat and human T19M were obtained by polymerase chain reaction (PCR) using oligonucleotide primers encoding the nucleotide substitution, Phusion high-fidelity DNA polymerase (New England Biolabs, Ipswich, MA) and plasmid templates containing wild-type human Cx46 in pSP64TII and pcDNA3.1/Hygro(+) (Invitrogen, Carlsbad, CA) or rat Cx46 in pBluescript (Stratagene, La Jolla, CA). Primers facing opposite directions (human Cx46T19M sense: **T**GGTCATCGGCAAGGTTTGGCTGACCGT and human Cx46T19M antisense: TGGAGTGCTCCTGTGCATTTTCTAAGAGTC; rat Cx46T19M sense: **T**GGTCATCGGCAAGGTGTGGCTGACCGTCC and rat Cx46T19M antisense: TAGAGTGCTCCTGCGCATTCTCCAGCAGCC) and spanning the DNA region encoding the mutated amino acid were designed to amplify the sequence of the full construct (including the vector sequence) according to the strategy used previously (Minogue et al. 2005); the plasmids were regenerated by re-ligation of the PCR product. Wild-type rat Cx46 and T19M were subsequently subcloned into pcDNA3.1(+) (Invitrogen) and PBI-CMV3 (Clontech). Rat Cx46T19M was subcloned into pSP64TII.

Plasmids were also produced that encoded human Cx46 or T19M fused to Enhanced Green Fluorescent Protein (EGFP), following a 19 amino acid linker sequence. The coding regions of human Cx46 or T19M were amplified by PCR using PfuTurbo DNA Polymerase (Stratagene) and sense primer: TCCGAATTCACTAGTGAGCCGCCATGGGCGACTGGAG, and antisense primer: CTAAGAATTCGATTTCCTCCGATGGCCAAGTCCTCCGG, then subcloned into the *Eco*RI site of the expression vector pEGFPN1 (Clontech Laboratories, Mountain View, CA).

The coding regions of all constructs were fully sequenced to ensure that PCR amplification did not introduce additional unwanted mutations.

### Cell Culture and Immunofluorescence

For the immunofluorescence experiments, HeLa cells were plated on 4-well chamber slides (LAB TEK, Nalge Nunc International, Naperville, IL) or glass coverslips, and grown as previously described (Berthoud et al. 2003; Tong et al. 2013). The cells were transiently transfected with wild-type Cx46 or T19M using Lipofectin Transfection Reagent (Invitrogen) and PLUS Reagent (Invitrogen). Eighteen to 48 h later, cells were fixed in 4 % paraformaldehyde. After fixation, cells were subjected to immunofluorescence using rabbit anti-Cx46 antibodies and Cy3-conjugated goat anti-rabbit IgG antibodies (Jackson ImmunoResearch, West Grove, PA) as previously described (Minogue et al. 2005). Cells were examined using a Zeiss Plan Apochromat 40× objective (n.a., 1.0) in an Axioplan 2 microscope (Carl Zeiss Inc., München, Germany) equipped with a mercury lamp, and images were acquired with a Zeiss AxioCam digital camera and Zeiss AxioVision software (Carl Zeiss Inc.). Figures were assembled using Adobe Photoshop CS3 Extended (Adobe Systems Inc., San Jose, CA).

### Uptake of Connexin-Permeant Tracers

The dye uptake experiments utilized a communication-deficient clone of HeLa cells provided by V.K. Verselis (Albert Einstein College of Medicine, Bronx, NY). They were transfected with a bidirectional promoter vector, PBI-CMV3, encoding rat Cx46 or T19M and the reporter protein, Zaza green, or human Cx46-EGFP or human T19M-EGFP using Happyfect (Mayflower Bioscience, St. Louis, MO). Cells were tested for dye uptake 1 day later by a 20-min exposure to sodium Ringer’s solution (150 mM sodium gluconate or NaCl, 4.7 mM KCl, 5 mM glucose, and 5 mM HEPES, pH 7.4) with or without divalent cations and to which 10 μM 4′,6-diamino-2-phenylindole dihydrochloride (DAPI) was added. Then, cells were washed in the sodium Ringer’s solution containing 1 mM Ca^2+^ and 1 mM Mg^2+^ without dyes and examined by epifluorescence using a Nikon Eclipse inverted microscope equipped with a CCD camera (Photometrics, Tucson, AZ) associated with image analysis software (NIS Elements AR 3.0, Nikon). Uptake of DAPI was quantified by placing a region of interest (ROI) over the nuclei of cells expressing the reporter protein and measuring the mean intensity of DAPI after correction for background fluorescence measured in a cell-free region. Two to five 20× fields of view were analyzed for each treatment to generate histograms of the intensity of DAPI in reporter protein-expressing cells. Dead cells identified by morphological criteria were excluded from the data analysis.

Time-lapse experiments using rat Cx46 or T19M were performed using a closed bath insert (RC-37FC; Warner Instruments) as previously described (Ebihara et al. 2011). Four μM DAPI was added to all of the perfusion solutions. The rate of DAPI uptake under control conditions, following removal of divalent cations, and after addition of La^3+^ was determined by fitting the time course of DAPI uptake to a linear regression.

### Expression of Connexins in Xenopus Oocytes

Connexin cRNAs were synthesized using the mMessage mMachine in vitro transcription kit (Ambion, Austin, TX) according to the manufacturer’s instructions. The amount of cRNA was quantitated by measuring the absorbance at 260 nm.

Adult female *Xenopus laevis* frogs were anesthetized with tricaine and a partial ovariectomy was performed in accordance with protocols approved by the Animal Care and Use Committee at Rosalind Franklin University in North Chicago, IL. The oocytes were manually defolliculated after treating them with collagenase IA (Worthington Biochemical Corporation, Lakewood, NJ). Stage V and VI oocytes were selected and pressure-injected using a Nanoject variable microinjection apparatus (model No. 3-000-203, Drummond Scientific, Broomal, PA) with 36.8 nl of 0.5–600 ng/μl of connexin cRNA and 5 ng/36.8 nl of oligonucleotides antisense to mRNA for *Xenopus* Cx38 as previously described (Ebihara 1996). The oocytes were incubated overnight at 18 °C in L-15 (GIBCO-Invitrogen, Carlsbad, CA) containing 2 mM CaCl_2_ prior to performing electrophysiological experiments.

### Electrophysiological Measurements

For measurement of gap junctional coupling, connexin cRNA-injected oocytes were devitellinized and paired as previously described (Ebihara 1992). Double two-microelectrode voltage clamp experiments were performed using GeneClamp 500 (Molecular Devices, Sunnyvale, CA) and a TEV-200A (Dagan Corporation, MN) as previously described (Xu and Ebihara 1999). Pulse generation and data acquisition were performed using a PC computer equipped with PCLAMP9 software and a Digidata 1322A data acquisition system (Molecular Devices). All experiments were performed at room temperature (20–22 °C).

Hemi-channel currents were recorded from single transfected HeLa cells using the whole cell variant of the patch clamp technique. The resistance of the patch pipettes was 2–4 MΩ when filled with standard internal solution. After rupturing the membrane patch, the series resistance was usually <10 MΩ and was therefore not compensated. The internal solution contained: 140 mM CsCl, 10 mM EGTA, 2 mM MgATP, 3 mM Na_2_ATP, 10 mM HEPES, pH 7.4. The standard external bath solution contained 150 mM Na gluconate, 4.7 mM KCl, 1 mM MgCl_2_, 1 mM CaCl_2_, 5 mM glucose, 5 mM HEPES, pH 7.4. The membrane potentials were not corrected for liquid-junction potentials.

## Results

**Fig. 1:**
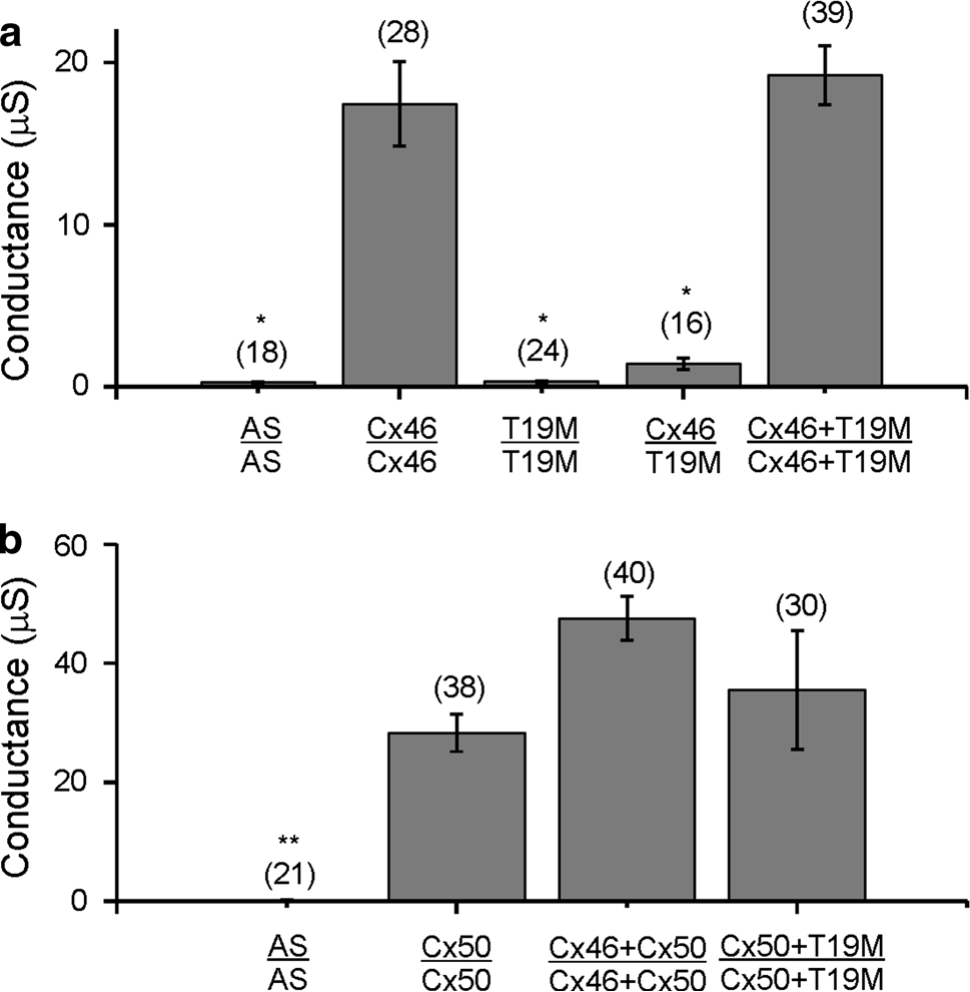
T19M does not induce gap junctional coupling when expressed by itself, and it acts as a loss-of-function mutation without dominant-negative inhibition when co-expressed with wild-type lens connexins. *Bar graphs* show mean gap junctional conductances in pairs of oocytes expressing different combinations of wild-type and mutant lens connexins as determined using the double two-electrode voltage clamp technique. **a** Rat Cx46 or T19M were expressed alone or in combination with each other. **b** Mouse Cx50 was expressed alone or in combination with either rat Cx46 or T19M. AS indicates oocytes that were injected with no cRNA (i.e., *Xenopus* Cx38 antisense oligonucleotide alone). The number of pairs tested is indicated within *parentheses*. *p < 0.001 (Student’s *t* test compared with Cx46-injected oocyte pairs); **p < 0.001 (Student’s *t* test compared with Cx50-injected oocyte pairs)

We initially tested whether T19M was able to induce gap junctional (intercellular) conductances in paired *Xenopus* oocytes using the double two-electrode voltage clamp technique. Because rodent Cx46 induces higher levels of gap junctional currents than human Cx46 in this system, experiments were initially performed after injecting cRNAs encoding either wild-type or mutant rat Cx46. Homotypic oocyte pairs expressing wild-type rat Cx46 were well coupled, but oocyte pairs injected with rat T19M cRNA showed no coupling above control levels (Fig. 1a). Furthermore, oocytes expressing rat T19M failed to induce significant coupling when paired heterotypically with oocytes expressing wild-type Cx46 (Fig. 1a).

To determine if co-expression of T19M affected the junctional conductance produced by wild-type lens connexins, we tested pairs of oocytes co-injected with equal amounts of cRNAs encoding rat T19M and wild-type rat Cx46 or mouse Cx50. The conductances induced in oocyte pairs co-expressing T19M with wild-type Cx46 did not differ significantly from those expressing only wild-type Cx46 (Fig. 1a). Similarly, the conductances induced in oocyte pairs co-expressing T19M with Cx50 did not differ significantly from those expressing Cx50 alone or those co-expressing wild-type Cx50 and wild-type Cx46 (Fig. 1b). These results suggest that T19M acts as a loss-of-function mutation without dominant-negative inhibition of intercellular communication.

**Fig. 2:**
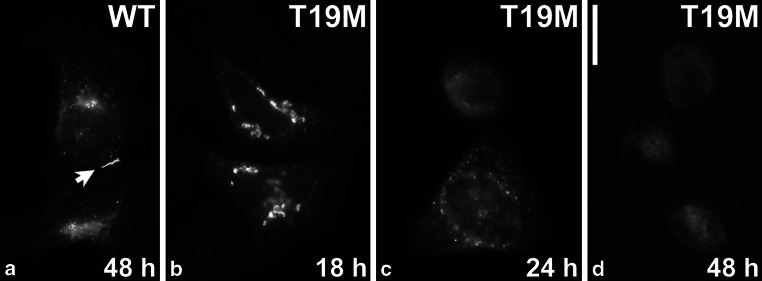
T19M is inefficient at forming gap junction plaques. Photomicrographs show the distribution of wild-type rat Cx46 (**a**) and T19M (**b**–**d**) at the indicated times following transient transfection of HeLa cells. *Bar* 30 μM

To determine the ability of T19M to form gap junction plaques, we performed immunofluorescence staining on transiently transfected HeLa cells. Cells transfected with wild-type rat Cx46 showed strong staining at gap junction plaques and some staining in the perinuclear region, likely corresponding to the Golgi apparatus (Fig. 2a). Surprisingly, in multiple independent experiments we detected very few cells containing immunoreactive Cx46 after transfection with rat T19M at our usual time of observation (48 h). Eighteen hours following transfection, most Cx46T19M-expressing cells showed a normal morphology with strong perinuclear staining and no gap junctional plaques (Fig. 2b). By 24 h after transfection, cells had started to round up and the abundance of cells expressing the Cx46 mutant was decreased (Fig. 2c). After 48–72 h, cells showed low intensity of Cx46T19M immunoreactivity, lacked detectable gap junctions, and were rounded up (Fig. 2d). The striking decline in numbers of T19M-expressing cells and their rounded morphology suggested that the mutated protein had toxic effects.

To assess the potential toxicity of T19M, we quantified the proportion of rounded up Zaza-green positive cells (expressed with the connexin using the bidirectional promoter vector, PBI-CMV3) after transfection. For these experiments, we used a different transfection reagent (Happyfect), because it has been reported to be less toxic to cultured cells. Seventy-two hours after transfection, 89 % (149/167) of the T19M transfected cells were rounded up as compared to only 14 % (21/126) of the wild-type Cx46 and 16 % (51/318) of vector-transfected cells. The morphology of the cells expressing T19M was strikingly different from that of the cells expressing wild-type Cx46 as illustrated in Fig. S1. In addition to rounding up, many of the T19M expressing cells showed extensive membrane blebbing which is often seen in dying cells.

**Fig. 3:**
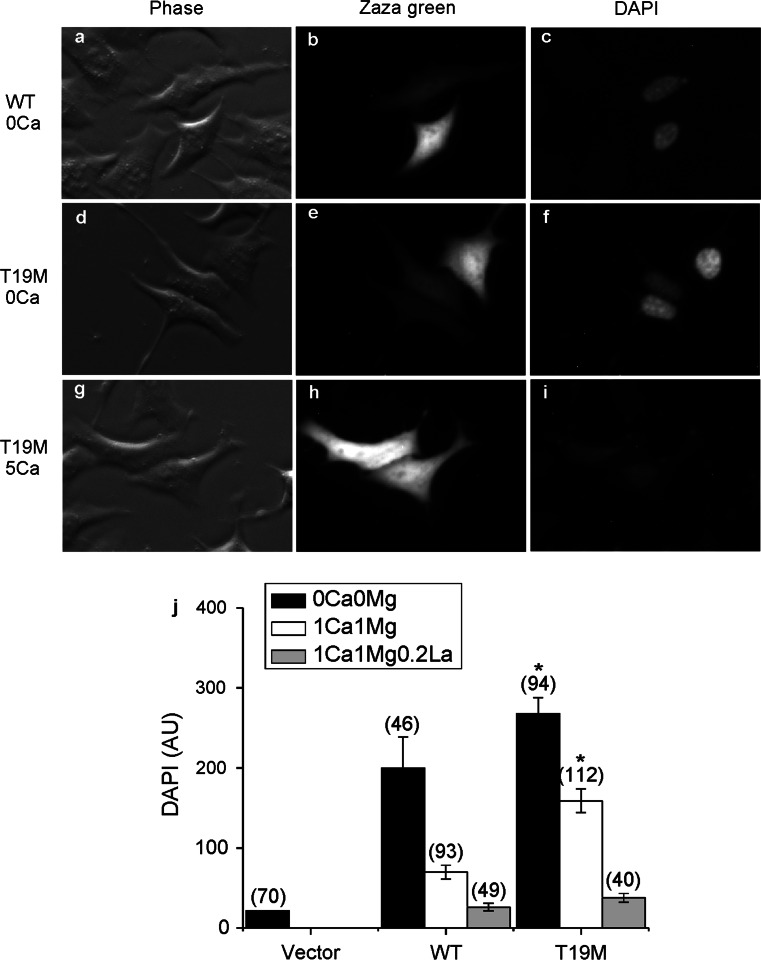
T19M causes increased uptake of connexon-permeant dyes. Photomicrographs show examples of HeLa cells that were transfected with wild-type rat Cx46 (**a**–**c**) or T19M (**d–i**) (using the vector PBI-CMV3 which also drives expression of Zaza green) and incubated a day later with DAPI in Na gluconate Ringer’s solution containing 0 mM Ca^2+^ (**a**–**f**) or 5 mM Ca^+2^ (**g**–**i**) for 20 min. Phase contrast images (**a**, **d**, **g**). Zaza-green fluorescence (**b**, **e**, **h**). DAPI fluorescence (**c**, **f**, **i**). After a 20-min incubation in control solution containing 0 mM Ca^2+^, cells expressing T19M showed DAPI uptake (**e**, **f**) that was mostly inhibited by 5 mM Ca^2+^ (**h**, **i**). *Bar graph* summarizes the quantification of the DAPI uptake data (**j**). Data are graphed as mean ± SEM. The number of cells tested is indicated within *parentheses*. *p < 0.001 (Mann–Whitney rank sum test compared with wild-type rat Cx46-transfected cells)

To test the hypothesis that the apparent cell toxicity in T19M-expressing cells was a consequence of aberrant T19M hemi-channel activity, we examined uptake of the connexon-permeant dye, DAPI, in transfected HeLa cells. Cells that expressed rat Cx46 or T19M were identified by the fluorescence of the reporter protein, Zaza green (Fig. 3). When incubated in the absence of divalent cations or in the presence of 1 mM external Ca^2+^ concentration ([Ca^2+^]_o_ and 1 mM [Mg^2+^]_o_), cells expressing T19M showed significantly increased DAPI uptake compared to cells expressing wild-type Cx46 or control (Zaza-green negative) cells. The T19M-induced dye uptake was mostly blocked by increasing the [Ca^+2^]_o_ to 5 mM.

In some experiments, we used propidium iodide (PI) and DAPI as the connexon-permeant tracers (data not shown). These experiments showed a linear relationship between the rates of DAPI and PI uptake. However, the rate of PI uptake was much smaller than the rate of DAPI uptake.

**Fig. 4:**
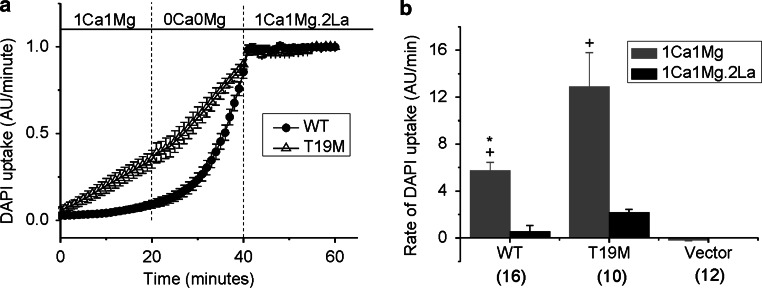
The rate of DAPI uptake is increased by lowering divalent cations and inhibited by La^3+^. Average time course of DAPI uptake by transfected HeLa cells in control solution (1 mM Ca^2+^, 1 mM Mg^2+^), in external solutions with no added divalent cations and in control solution plus 200 µM La^3+^. **a** Wild-type rat Cx46 (*closed circles*); T19M (*open triangles*). To measure changes in the rate of dye uptake over time, the mean DAPI fluorescence intensity per pixel from ROI’s located in the nuclei of Zaza-green positive cells were normalized to mean DAPI fluorescence intensity of the ROI’s at 60 min, averaged and plotted as a function of time. The cells were initially bathed in control solution (containing 1 mM Ca^2+^, 1 mM Mg^2+^). Then, the cells were exposed to a solution containing no added divalent cations followed by reperfusion with control solution containing 200 μM La^3+^. All the solutions contained 4 μM DAPI. **b** *Bar graph* shows the rates of DAPI uptake in cells expressing wild-type Cx46, T19M, or vector alone in the presence of 1 mM Ca^2+^, 1 mM Mg^2+^ (*gray bar*); or 1 mM Ca^2+^, 1 mM Mg^2+^, 0.2 mM La^3+^ (*black bar*). Data are presented as the mean ± SEM. *p < 0.002 (Mann–Whitney rank sum test compared with T19M-transfected cells); ^+^ *p* < 0.001 (Mann–Whitney rank sum test compared with vector-transfected cells). The number of cells analyzed is indicated within *parentheses*

To examine the effect of wild-type and mutant Cx46 on hemi-channel activity in greater detail, we measured the uptake of DAPI by time-lapse recordings in HeLa cells expressing wild-type rat Cx46, T19M, or vector alone (Fig. 4). In the presence of control external solution (containing 1 mM Ca^2+^and 1 mM Mg^2+^), cells expressing T19M showed significantly higher rates of DAPI uptake than those expressing wild-type Cx46 or vector alone. After changing the bathing medium to a solution free of divalent cations, the rate of DAPI uptake increased by approximately twofold in the T19M cells and by up to 19-fold in the Cx46 cells. Little or no increase in dye uptake was observed in control cells expressing vector alone (data not shown). Dye uptake was completely blocked by application of 200 μM lanthanum, a nonspecific connexin hemi-channel blocker. Dye uptake was also ~90 % blocked in Cx46 cells and 77 % blocked in T19M cells by application of 500 μM carbenoxolone. These results suggest that the T19M channels are less sensitive to blockade by divalent cations than wild-type Cx46 hemi-channels. Interestingly, the effect of divalent cation-free conditions on the wild-type Cx46 cells was slow, requiring greater than 15 min to reach a new steady-state. In contrast, the effect of divalent cation-free conditions on the T19M cells was relatively fast, reaching steady-state in <2 min.

**Fig. 5:**
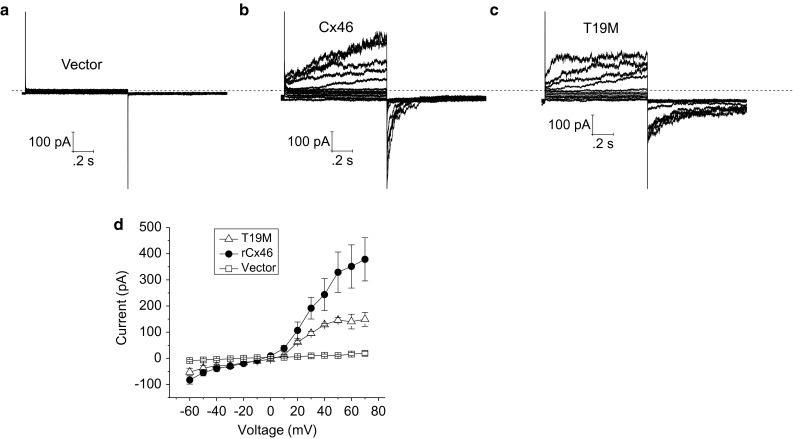
Representative families of current traces recorded from single HeLa cells transfected with vector alone (**a**), wild-type rat Cx46 (**b**), or T19M (**c**). Families of current traces were recorded in response to a series of voltage clamp steps between −60 and 50 mV in increments of 10 mV from a holding potential of −60 mV. *Dashed line* indicates zero current level. **d** Average steady-state I–V relationships for vector alone (*open squares*, *n* = 5), wild-type (*solid circles*, *n* = 4), and T19M (*open triangles*, *n* = 3)

**Fig. 6:**
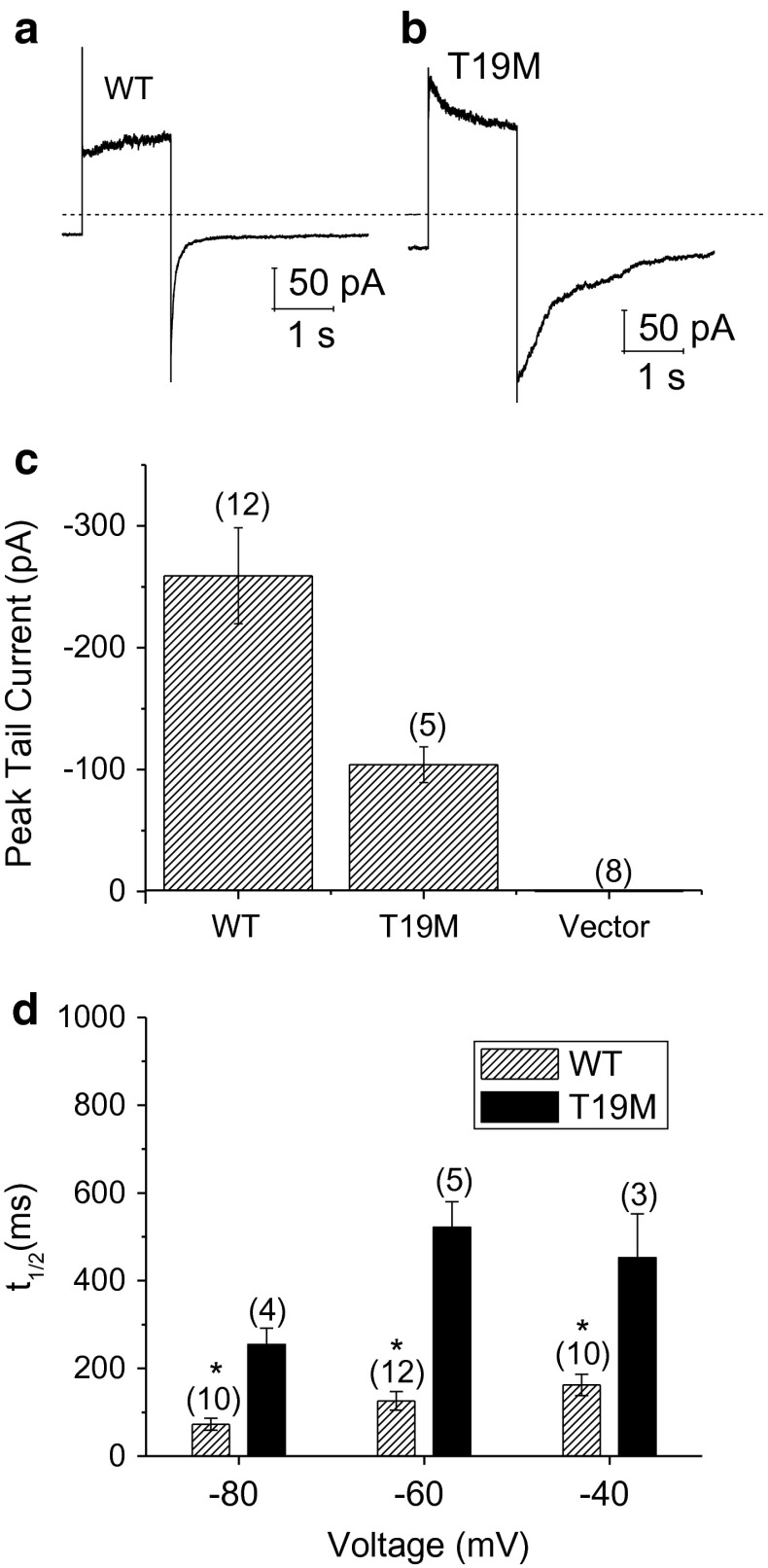
T19M hemi-channels show alterations in voltage gating. Ensemble averaged current traces recorded from cells expressing wild-type rat Cx46 (**a**) or T19M (**b**) in response to a 2-s voltage clamp step to 80 mV followed by a hyperpolarizing step to −60 mV. The holding potential was −60 mV. *Dashed line* indicates zero current level. **c** Averaged peak tail currents at −60 mV. The number of cells tested is indicated within *parentheses*. **d** *Bar graph* summarizes the *t*_1/2_’s of deactivation of peak tail currents for wild-type rat Cx46 (*hatched bars*) and T19M (*black bars*) at −80, −60, and −40 mV. Data are graphed as mean ± SEM. *p < 0.01 (Student’s *t* test or Mann–Whitney rank sum test compared with T19M-transfected cells). The number of cells analyzed is indicated within *parentheses*

To determine the effects of T19M on plasma membrane conductance of HeLa cells, whole cell patch clamp experiments were performed with cesium in the pipette and sodium in the bath. Figure 5 compares representative families of current traces (and averaged steady-state I–V relationships) recorded from HeLa cells expressing Cx46, T19M, or Zaza green alone in the presence of 1 mM [Ca^+2^]_o_ and 1 mM [Mg^+2^]_o_. Both Cx46- and T19M-expressing cells exhibited currents that were mostly closed at −60 mV and activated in response to depolarizing voltage clamp steps in a time- and voltage-dependent manner. These currents were not observed in either non-transfected cells or Zaza-green-transfected cells, indicating that they could be attributed to Cx46 and T19M hemi-channels. Both wild-type and mutant currents could be readily observed in cells transfected with similar amounts of DNA even when the bath solution contained 1 mM Ca^2+^ (and 1 mM Mg^2+^). However, the T19M mutation altered the kinetics of channel gating. T19M currents activated more rapidly and had a more pronounced and prolonged inactivation phase than the wild-type Cx46 currents at large positive potentials as illustrated in Fig. 6a and b. In addition, the time course of deactivation of the T19M currents at negative potentials was slower than that of wild-type Cx46. This effect was quantified by measuring the time required for the tail current to decay to 50 % of its peak value (*t*_1/2_). Over the transmembrane voltage (*V*_m_) range between −80 and −40 mV, the average *t*_1/2_ values were significantly longer for T19M than for wild-type Cx46 (Fig. 6d). This effect did not appear to correlate with changes in current density since the amplitudes of the T19M tail currents used in the data analysis were smaller than those of the WT tail currents (Fig. 6c).

**Fig. 7:**
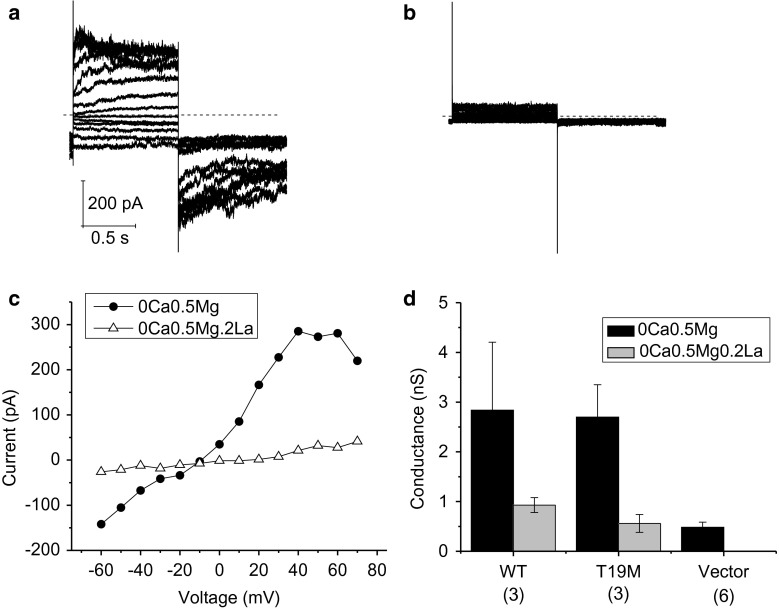
Effect of lanthanum ions. Currents before (**a**) and after the application of La^3+^ (**b**; 200 µM) recorded from a HeLa cell expressing T19M. Families of current traces were recorded in response to a series of voltage clamp steps between −60 and 70 mV in increments of 10 mV from a holding potential of −60 mV. *Dashed line* indicates zero current level. **c** I–V relations obtained from the data shown in (**a**, **b**). The current was measured at the end of the 1-s pulse and plotted as a function of voltage. The concentrations of divalent cations in the bath solution were reduced to zero added Ca^2+^ and 0.5 mM Mg^2+^ to augment the size of the hemi-channel currents. **d** *Bar graph* summarizes the input conductance measured at −60 mV in HeLa cells expressing wild-type Cx46, T19M, or vector (*alone*) when exposed to extracellular solutions containing 0 mM Ca^2+^, 0.5 mM Mg^2+^ (*black bars*) or 0 mM Ca^2+^, 0.5 mM Mg^2+^, 0.2 mM La^3+^ (*gray bars*). The number of cells analyzed is indicated within *parentheses*

To determine if the small, persistent inward current observed at negative potentials in T19M-expressing cells was due to hemi-channels, we used the nonspecific hemi-channel blocker, La^+3^ (John et al. 1999; Contreras et al. 2002). Application of 200 μM La^3+^ blocked most of the time- and voltage-dependent component of the current (Fig. 7). It also reduced the inward current at the holding potential and decreased the cell input conductance to values comparable to those observed in vector-transfected control cells. Similar results were obtained for wild-type Cx46. The Cx46 hemi-channel currents could also be partially blocked by 200 μM carbenoxolone. These findings suggest that a small number of Cx46 hemi-channels remain open even in the presence of divalent cations and that these channels can account for the dye uptake observed in the presence of divalent cations.

**Fig. 8:**
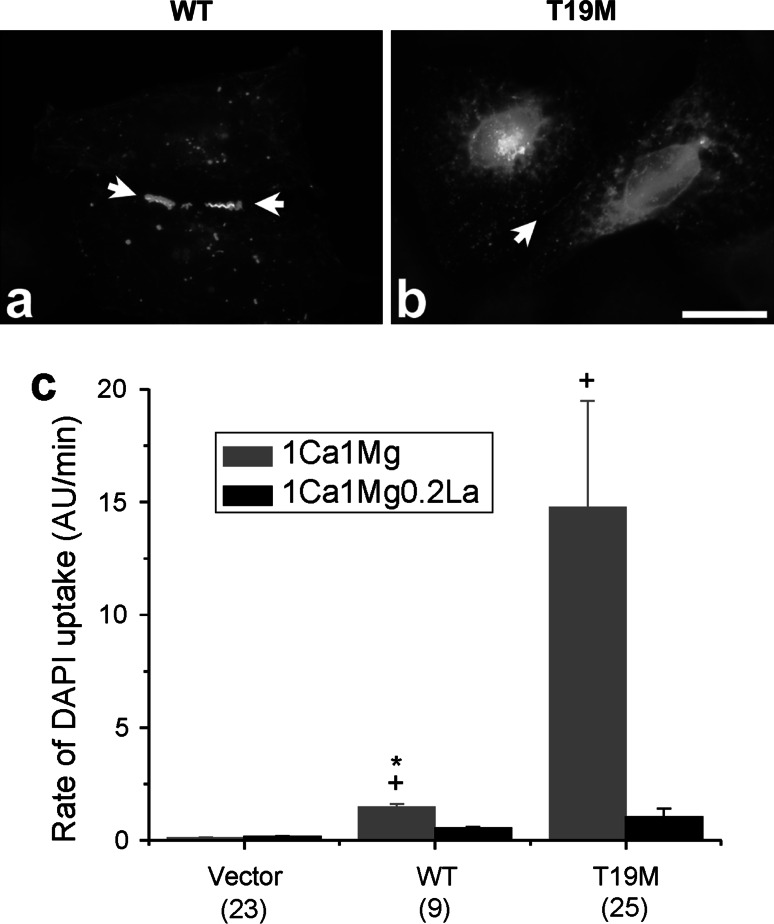
Effects of T19M mutation on human Cx46. Photomicrographs showing the distribution of human wild-type Cx46 (**a**) and T19M (**b**) in transfected HeLa cells. *Bar* 30 µm. **c** *Bar graph* summarizes the DAPI uptake data for vector alone, human wild-type Cx46 and T19M obtained in transfected HeLa cells when exposed to extracellular solutions containing 1 mM Ca^2+^, 1 mM Mg^2+^ (*gray bars*) or 1 mM Ca^2+^, 1 mM Mg^2+^, 0.2 mM La^3+^ (*black bars*). Data are graphed as mean ± SEM. *p < 0.004 (Mann–Whitney rank sum test compared with T19M-transfected cells in the same external solution); ^+^ *p* < 0.001 (Mann–Whitney rank sum test compared with vector-transfected cells). The number of cells analyzed is indicated within *parentheses*

Because the T19M was originally identified in a human family (Santhiya et al. 2010), we investigated whether human wild-type Cx46 and T19M behaved similarly. Indeed, wild-type human Cx46 localized extensively to gap junction plaques with some localization in the cytoplasm in transfected HeLa cells (Fig. 8a). In contrast, human T19M immunoreactivity was detected mainly in intracellular compartments; on very rare occasions a faint staining at appositional membranes was detected (Fig. 8b).

Similar to the results obtained with the rat constructs, HeLa cells expressing human T19M-EGFP showed much higher rates of DAPI uptake in the presence of control external solutions (containing 1 mM Ca^2+^ and 1 mM Mg^2+^) than cells expressing wild-type Cx46 (Fig. 8c). The dye uptake in cells expressing either human Cx46 or T19M was completely blocked by application of 200 µM La^3+^ (Fig. 8c).

Thus, the T19M mutation disrupted gap junctional plaque formation and extracellular divalent cation-dependent regulation of hemi-channel gating regardless of the species of origin of the Cx46.

## Discussion

In this paper, we have shown that mutation of the conserved threonine-19 to methionine causes a unique spectrum of alterations of connexin behavior. This residue is important for both cell biological and physiological functions, since Cx46T19M exhibits impaired gap junction assembly and abnormal gap junctional channel and hemi-channel activities.

The inability of T19M to support intercellular communication is likely due to its poor formation of gap junction plaques. The absence of gap junctions could potentially result from altered trafficking of the connexin or reduced assembly of connexons into immunodetectable gap junction plaques. The detection of T19M hemi-channel activity by electrophysiology and dye uptake implies that the mutant forms oligomers that traffic properly to the plasma membrane. Therefore, the most likely explanation is that the mutant protein is inefficient at assembling into gap junction plaques. Several other connexin mutants that cause cataracts (including Cx50D47N and Cx50R23T) (Arora et al. 2008; Thomas et al. 2008) or other diseases (Di et al. 2002; Marziano et al. 2003; VanSlyke et al. 2000) lack gap junction function because of inefficient formation of gap junction plaques ascribed to impaired trafficking of the connexin. In contrast, T19M has impaired formation of gap junction plaques, but it can traffic to the plasma membrane.

In our studies, T19M did not cause significant inhibition of co-expressed wild-type Cx46 or Cx50 gap junction activity. This loss-of-function behavior for gap junctional intercellular communication is similar to that reported for some other cataract-linked connexin mutants (e.g., Cx50D47N, Cx46N63S, Cx46fs380) (Arora et al. 2008; Pal et al. 2000), but contrasts with the dominant-negative effect induced by other mutants (e.g., Cx50P88S, Cx50P88Q) (Arora et al. 2006; Pal et al. 1999). The behavior of T19M suggests that either it does not co-oligomerize with wild-type Cx46 or Cx50 or that its presence in mixed gap junction channels has no significant effect on function.

T19M also showed enhanced hemi-channel activity in the presence of physiological concentrations of divalent cations. A “gain of hemi-channel function” has been reported for some cataract-associated connexin mutants including Cx50G46V (Minogue et al. 2009; Tong et al. 2013) and Cx46G143R (Ren et al. 2013); however, unlike Cx46T19M, these mutants form gap junction plaques and support high levels of intercellular communication. A few Cx43 mutants that form non-functional gap junction plaques have been reported to form functional hemi-channels based on ATP release (Dobrowolski et al. 2007). Some Cx26 mutants associated with keratitis-ichthyosis-deafness syndrome display increased hemi-channel activity, but do not form gap junction plaques (Mhaske et al. 2013). There are a number of possible mechanisms that could lead to increased hemi-channel activity. In the case of T19M, the impaired hemi-channel closing at negative potentials and the reduced sensitivity to external calcium implies that dysregulation of T19M hemi-channel gating (loop gating; Bukauskas et al. 1995) is the mechanism responsible for increased hemi-channel activity. Even the presence of a few functional T19M hemi-channels on the cell surface might be sufficient to account for the increase in hemi-channel activity.

Previous studies have shown that the N-terminus of Cx46 contributes to the pore lining and plays a critical role in channel gating (Kronengold et al. 2003; Tong and Ebihara 2006; Trexler et al. 2000). Homology models of Cx46 based on the crystal structure of Cx26 indicate that residue T19 lies on the cytoplasmic side of Cx46 near the hinge region where the N-terminus bends into the pore. Replacement of R9 in the N-terminus of the chicken ortholog of Cx46 changes voltage-dependent hemi-channel gating or loop gating (Tong and Ebihara 2006). Replacement of T19 by methionine may cause long distance conformational changes in the more proximal portion of the N-terminus resulting in alterations in channel gating. Long-range conformational changes might affect the extracellular loops, because these regions are important for processes that are altered in the T19M mutant including loop gating and hemi-channel docking.

The various defects in Cx46 function caused by the T19M mutation may all contribute to the development of cataracts in people expressing the mutant and the dominant inheritance pattern. Homeostasis in the lens is normally supported by a circulation of water and ions that requires gap junction intercellular communication (Mathias et al. 2010). Because absence of one wild-type Cx46 allele in heterozygous mice decreases coupling conductance by 25 % in differentiating fiber cells and by 50 % in mature fiber cells (Mathias et al. 2010), expression of T19M in the lens is expected to cause a reduction in the gap junction-mediated efflux of water, ions, and other solutes to the surface of the lens. However, haplodeficiency for a lens fiber connexin is not sufficient to cause cataracts in Cx46- and Cx50-null mice (Gong et al. 1997; Rong et al. 2002; White et al. 1998). Thus, it is likely that additional toxic effects contribute to the pathogenicity of T19M in people and underlie the dominant inheritance of cataracts, most likely through the gain of hemi-channel activity. This would cause an increased influx of sodium and calcium into the fiber cells, contributing to their intracellular accumulation (especially since their efflux to the surface of the lens through a pathway involving gap junctions is reduced). These alterations could lead to the loss of metabolic homeostasis, activation of various enzymes (including proteases), and cataract formation. The cataracts in people carrying CX46T19M were described as “posterior polar” (Santhiya et al. 2010). Unfortunately, the mechanism for development of this type of cataract is not currently understood (Kalantan 2012). Moreover, there is no clear relationship between a specific lens connexin mutation and the cataract phenotype; indeed, the same mutation can produce cataracts with different appearances (reviewed in Beyer et al. 2013).

In conclusion, our data show that the Cx46 mutation, T19M, is inefficient at forming gap junction plaques, does not induce intercellular communication, and causes increased hemi-channel activity. This rather unique spectrum of defects demonstrates that T19 is a critical amino acid within the N-terminus of Cx46. Because the identity of this amino acid is highly conserved among connexin subtypes, it is likely that mutations at this position would also affect the function of other connexins and contribute to disease in other tissues.

## Electronic supplementary material

Below is the link to the electronic supplementary material.

Supplementary material 1 (PDF 153 kb)
