# Supplementary material for: The Connexin46 Mutant, Cx46T19M, Causes Loss of Gap Junction Function and Alters Hemi-channel Gating
Source: J Membr Biol. 2014 Nov 18;248(1):145–55. doi: 10.1007/s00232-014-9752-y (PMC4300453; doi:10.1007/s00232-014-9752-y)
Supplement: Supplementary file 1 — Supplementary material 1 (PDF 153 kb) [file 232_2014_9752_MOESM1_ESM.pdf]

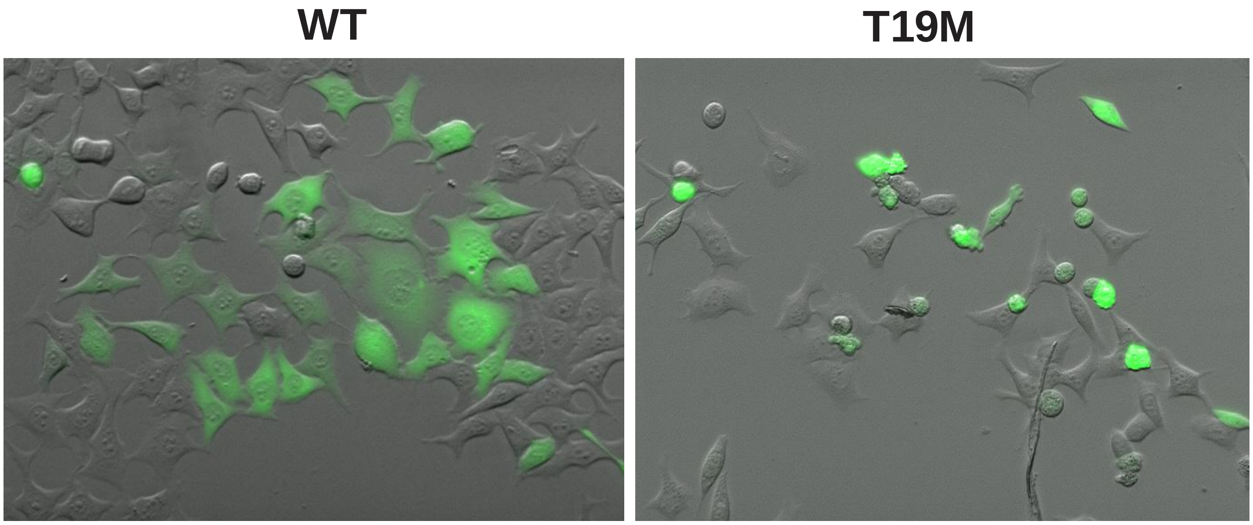

Supplemental Fig 1 Photomicrographs show the morphology of HeLa cells expressing wild-type Cx46 and T19M at seventy two hours after transfection. The morphology of the cells expressing T19M was strikingly different than the morphology of the cells expressing wild-type Cx46. In addition to rounding up, many of the T19M expressing cells showed extensive membrane blebbing which is often seen in dying cells.

The connexin46 mutant Cx46T19M, causes loss of gap junctional function and alters hemichannel gating, Journal of Membrane Biology, Tong JJ, Minogue PJ, Kobeszko M, Beyer EC, Berthoud VM, Ebihara L
Email:lisa.ebihara@rosalindfranklin.edu
